# Stationary Computed Tomography for Space and other Resource-constrained Environments

**DOI:** 10.1038/s41598-018-32505-z

**Published:** 2018-09-21

**Authors:** Avilash Cramer, Jake Hecla, Dufan Wu, Xiaochun Lai, Tim Boers, Kai Yang, Tim Moulton, Steven Kenyon, Zaven Arzoumanian, Wolfgang Krull, Keith Gendreau, Rajiv Gupta

**Affiliations:** 1 Massachusetts Institute of Technology, Cambridge, 02139 USA; 2 Harvard Medical School, Boston, 20115 USA; 3 Massachusetts General Hospital, Boston, 02114 USA; 4 Consortia for Improving Medicine with Innovative Technology, Boston, 02114 USA; 5 University of Twente, Enschede, 7522 NB Netherlands; 6 NASA’s Goddard Space Flight Center, Greenbelt, 02771 USA

## Abstract

Computed tomography (CT) is used to diagnose many emergent medical conditions, including stroke and traumatic brain injuries. Unfortunately, the size, weight, and expense of CT systems make them largely inaccessible for patients outside of major hospitals. We have designed a module containing multiple miniature x-ray sources that could allow for CT systems to be significantly lighter, smaller, and cheaper, and to operate without any moving parts. We have developed a novel photocathode-based x-ray source, created by depositing a thin film of magnesium on an electron multiplier. When illuminated by a UV LED, this photocathode emits a beam of electrons, with a beam current of up to 1 mA. The produced electrons are accelerated through a high voltage to a tungsten target. These sources are individually addressable and can be pulsed rapidly, through electronic control of the LEDs. Seven of these sources are housed together in a 17.5 degree arc within a custom vacuum manifold. A full ring of these modules could be used for CT imaging. By pulsing the sources in series, we are able to demonstrate x-ray tomosynthesis without any moving parts. With a clinical flat-panel detector, we demonstrate 3D acquisition and reconstructions of a cadaver swine lung.

## Introduction

Roentgen’s discovery of “A new kind of ray” or x-ray (“for the sake of brevity”) ushered a new field of medical imaging that is still continuing to advance at a rapid pace^1^. X-ray computed tomography (CT), is a volumetric imaging technique in which a tomographic image is reconstructed from hundreds of projection x-ray images taken at different angles around the subject. It is the first-line imaging modality for diagnosing a wide variety of illnesses and injuries^2–5^. Modern CT scanners employ one or two x-ray sources mounted on a rotating gantry. As the gantry revolves around the patient (at speeds up to 300 revolutions per minute), the system acquires x-ray projections from multiple angles that are processed to generate a volumetric image^6,7^. The rotating gantry mechanism represents a considerable portion of the mass, size, and power requirements of a conventional CT scanner, hindering its application in resource-constrained environments; the rotating system’s large net angular momentum further reduces its suitability for use on board a spacecraft^8–10^. We describe a non-rotating CT system composed of multiple photocathode-driven x-ray modules that can be assembled into a ring or other geometry for tomographic imaging.

CT is used in the management of many injuries and diseases, such as aortic dissection, pericardial tamponade, cancers, and many others. Two classes of emergent conditions where CT is particularly vital are strokes and traumatic brain injuries (TBI).

As per the stroke management guidelines^2^ at the Massachusetts General Hospital, patients with symptoms indicative of a stroke first receive a non-contrast CT to determine if the stroke is haemorrhagic or ischemic, and to rule out a non-stroke pathology (a ‘mimic’, such as hypo/hyperglycemia, epilepsy, multiple sclerosis, and intracranial tumors). In the case that the stroke is ischemic, patients receive an intravenous contrast injection, and CT angiography is performed. This is done to visualize the occlusion in the artery, and the extent of the affected arterial territory. Such advanced imaging is performed to determine the extent of tissue death, and to determine whether a given patient is a candidate for more invasive, mechanical removal of a thrombus/embolus. Variations of this protocol are used in hospitals around the world^11–15^. Given the importance of CT in stroke management, and the widespread prevalence of strokes, a CT scanner that is portable and can be deployed in a variety of clinical settings is highly desirable. Such a scanner would allow doctors to distinguish between haemorrhagic and ischemic strokes, and in the latter case, deliver thrombolytic drugs in the field.

CT also plays a large role in clinical management of TBI. In the first 24 hours following a head injury - and if imaging is indicated - CT is the preferred neuroimaging technique. CT is the best imaging tool for detecting skull fractures, various forms of intracranial hemorrhages, and other rapidly emergent conditions that may require immediate intervention^3,16^.

However, even in high-income nations, CT systems are generally only available in major trauma centers and hospitals, and need to be supplied with continuous, 3-phase power. This lack of portability not only makes advanced healthcare delivery in rural communities challenging, it affects pre-hospital care in essentially all demographics. Worldwide, some three-quarters of the global population has no access to medical radiography of any kind, let alone computed tomography. The disparities in access for volumetric imaging are even more acute: in OECD countries, there is an average of one CT scanner per 65,000 people, while in low-income countries (defined as having a per capita Gross National Income of less than $1005), there is one CT scanner per 3,500,000 people^17^.

In the military sector, aircraft carriers, mobile forward surgical teams, and forward operating bases, generally operate without a volumetric medical imaging capability, despite performing surgeries that would routinely be accompanied by volumetric imaging in the civilian world^4,18,19^ (during the Iraq war, one mobile forward surgical team in Mosul was occasionally equipped with a single CT system). A CT system that is lightweight and modular - and can be broken down into man-portable components and assembled in the field - might be able to bring volumetric imaging closer to the battlefield.

Extended space missions represent another use case for a portable tomographic imaging system. A manned mission to Mars, for example, will take hundreds of days at the minimum^8^, and having advanced radiographic imaging capabilities could be a substantial benefit for the crew members of such a mission. Currently, radiographic imaging on the International Space Station is limited to ultrasound^10^. Unfortunately, a conventional CT system is not an option, due to its weight and the fact that a rotating gantry would impart an equal and opposite torque upon the spacecraft. A lightweight, motion-free, modular tomographic imaging system could address these issues.

## Results

**Figure 1:**
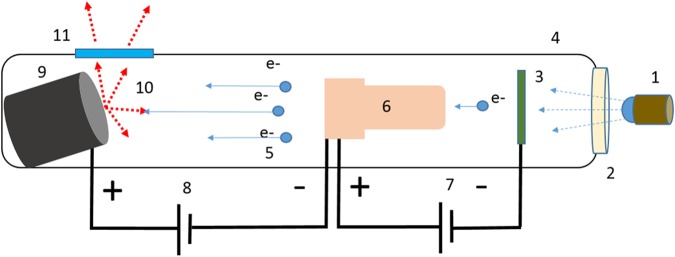
Schematic of the miniature x-ray source. A pulsable UV LED (1) emits UV photons which pass through a quartz window (2) into the vacuum manifold (4) and interact with a photoemissive magnesium film (3). This interaction produces electrons which are amplified by a Channeltron device (6), which is supplied with a 3 kV bias voltage (7). The amplified electrons (5) are accelerated through a large electric field provided by an external high voltage source (8), and impact on an angled tungsten target (9). This interaction (10) produces x-ray photons which leave the vacuum manifold through a beryllium window (11).

We demonstrate an x-ray source module consisting of a set of seven x-ray elements that can be turned on or off rapidly, in a programmable fashion, for distributed generation of x-rays from multiple points in space without any mechanical motion. The x-ray elements in this source module are miniature x-ray sources that are triggered by ultraviolet light. The UV light shining on a photocathode generates a small number of electrons by photo-emission. As depicted in Fig. 1, the photo-electrons are amplified using a high-gain electron multiplier and accelerated through a high voltage to a tungsten target. In the following subsections, we describe each of these components of our distributed x-ray source that enables multi-source motion-free tomography. Salient features of this design, as illustrated by various characterization experiments that we have conducted, are then described. The results of tomographic image reconstruction are illustrated with cadaveric specimens. Finally, we discuss what it would take to scale our prototype scanner to full-body human scanning.

### Electron Production

In conventional x-ray tubes, electrons are generated by heating a tungsten filament by running current through it. While thermionic electron sources can reliably produce a large electron flux (up to 1 ampere per square centimetre), they are large, have substantial power requirements, and cannot be pulsed on and off quickly due to their thermal inertia. A number of alternative x-ray generation arrangements have been proposed and are briefly described below in order to motivate the design choices made in our prototype.

Field emission is one alternative to thermionic electron generation. In particular, carbon nanotube (CNT) field emission x-ray sources have been demonstrated since the 1990s^20^. Non-rotating CT systems using carbon nanotube field emission sources have been proposed^21,22^.

CNT sources can have exceedingly small spot sizes, allowing for very high resolution x-ray imaging. However, CNT sources do have a number of challenges, including source stability^23^, reliability^24,25^, and most significantly, a complex manufacturing process. CNT sources are destroyed by even a brief exposure to pressures above a high vacuum. Because of these difficulties, few, if any, miniaturized, pulseable CNT-based field emission sources have been brought to market^26^.

Another alternative technique of electron generation is to use a photocathode. In photocathode emission, an incoming UV photon promotes an electron from the valence band on the photoemissive material into the conduction band. If the electron has enough energy to overcome the work function *ψ* of the photoemissive material, it can be ejected as a free electron.

In the proposed design, we use a metallic photocathode as it has several advantages for our particular application. While semiconductor and bi-alkalide photocathodes offer better quantum efficiency (QE) in the UV range than metallic photocathodes, they are generally difficult to produce, are intolerant of pressures greater than 10^−9^ Torr, and are degraded by exposure to air^27,28^. By contrast, metallic photocathodes are robust, easy to deposit, have a long lifetime, can survive being briefly brought up to atmospheric pressure, are intrinsically radiation hard, and feature a sub-picosecond response time for fast pulsing.

We used magnesium as our photocathode material illuminated by an LED emitting ~255 nm UV light. Magnesium has a relatively high quantum efficiency (QE) at the chosen UV wavelength, is very abundant, and has relatively few special handling concerns^27–29^. A magnesium photocathode will slowly absorb oxygen, and oxidize into MgO. This process can occur even in low vacuum. Although in special circumstances this oxidation can actually increase the quantum efficiency of a magnesium photocathode, in general, it will reduce it. However, the oxidation process is fortunately self-limiting and occurs over a course of years even at atmospheric pressure, and much more slowly in vacuum^30–32^.

Unlike CNT-based sources, the proposed distributed x-ray source does not require ultra-high vacuum. For example, we have operated it at 10^−6^ to 10^−7^ Torr for over one year without any cathode failure. Our cathode can even survive exposure to full atmospheric pressure for short durations, allowing for easy repair and replacement within the housing. By contrast, a CNT source would be irreparably damaged if it were exposed to even low (10^−7^ Torr) vacuum. For our source, a turbopump is sufficient to maintain vacuum; a diffusion pump or an ion pump, which are more complex and less portable, are not required.

### Electron Amplification

Metallic photocathodes produce too weak a photocurrent to be directly used for human-scale CT. Human imaging typically requires hundreds of milliamperes of x-ray tube current while a photocathode can generally produce only a fraction of a microampere. To amplify the photocurrent, we used a Channeltron^*TM*^ electron multiplier (Magnum 5900) with an adjustable bias voltage of 2500 to 4000 V. Originally developed for mass spectrometry, a Channeltron consists of a set of tightly wound spiral capillaries made of glass. The capillaries are coated with an emissive layer and supplied with a bias voltage that propels the injected electrons forward. The electrons produced by photoemission are injected into the input end of these capillaries. They bounce repeatedly from the walls of the Channeltron, with each interaction producing more electrons, before they exit from the output end. We constructed a photocathode by depositing a thin layer of magnesium on the input window of the Channeltron using a thermal evaporator (Denton Vacuum systems, 505-A). The deposition process, described in greater detail in the Methods section, is simple, inexpensive, and takes less than one hour.

The Channeltron amplifies the current produced by the photocathode on its input surface by a factor of up to 10^8^. As a result, the individual source elements can produce up to 1 mA of tube current. This tube current can be modulated by changing the bias voltage or input illumination of the Channeltron. Since the Channeltron is inside the vacuum manifold, programmable UV light stimulus to the photocathode at the input window of the Channeltron is provided by a 255 nm UV LED (Thorlabs Ball Lens LED250J) through a quartz window.

### X-ray Production

In conventional x-ray tubes, and in the distributed x-ray source proposed here, electrons are accelerated as they fall down the voltage gradient between the cathode and the anode. Typical accelerations voltages vary from a few thousand to millions of volts, depending on the application and the desired x-ray energies. Medical x-ray tomography typically uses tube voltages between 22 kVp (for digital breast tomosynthesis) and 140 kVp (such as in pelvic CT). Common target materials for medical imaging include tungsten, molybdenum, and rhodium. Accelerated electrons striking a metal target will emit x-rays over a range of energies dependent on the energy of the incident electrons and the energy levels of various electron shells of the target material. In general, this spectrum consists of a Bremsstrahlung background with superimposed emission line spectra of the anode material.

In the implemented design, an adjustable high positive voltage, up to 35–40 kVp, is applied to an anode target made of tungsten. The electrostatic field created by the positively charged anode accelerates the electrons from the output of each Channeltron, which is grounded. X-rays are generated as the high-energy electrons strike the anode. Because the heat is deposited at multiple anode targets, there is no need to physically rotate the individual targets for cooling.

### Source Module and Vacuum Manifold

**Figure 2:**
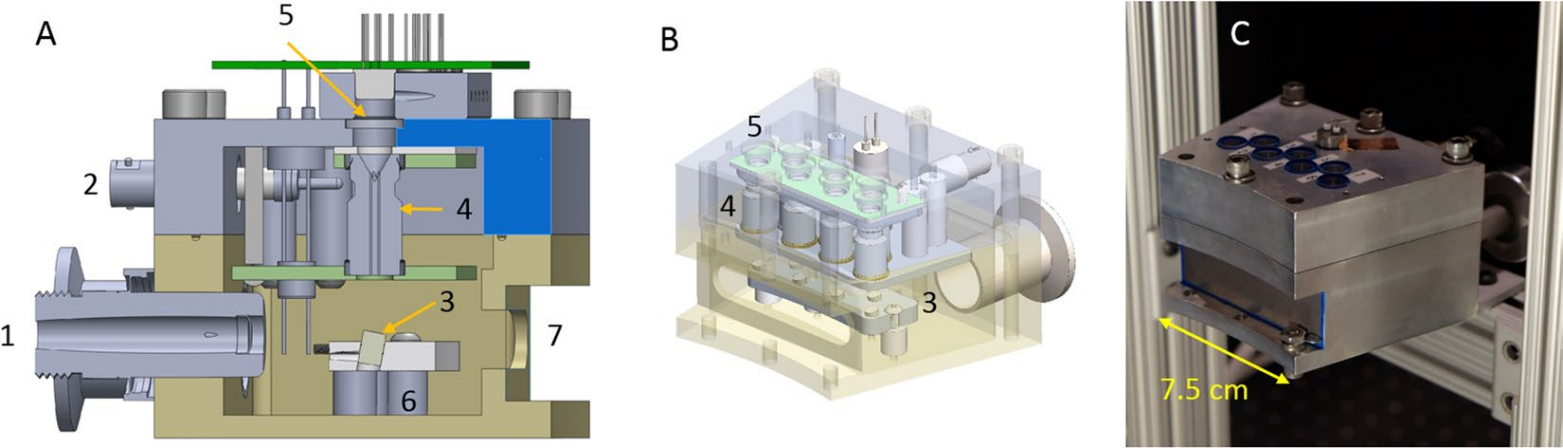
(**A**) Cut-through CAD model of the module. (**B**) Isometric view with a transparent outer housing. (**C**) Completed x-ray module with a beryllium sheet covering the x-ray window.(1) Vacuum connector (2) 3 kV bias line (3) tungsten targets (4) Channeltron electron amplifiers (5) quartz windows (6) High-voltage anode plate (7) x-ray window.

**Figure 3:**
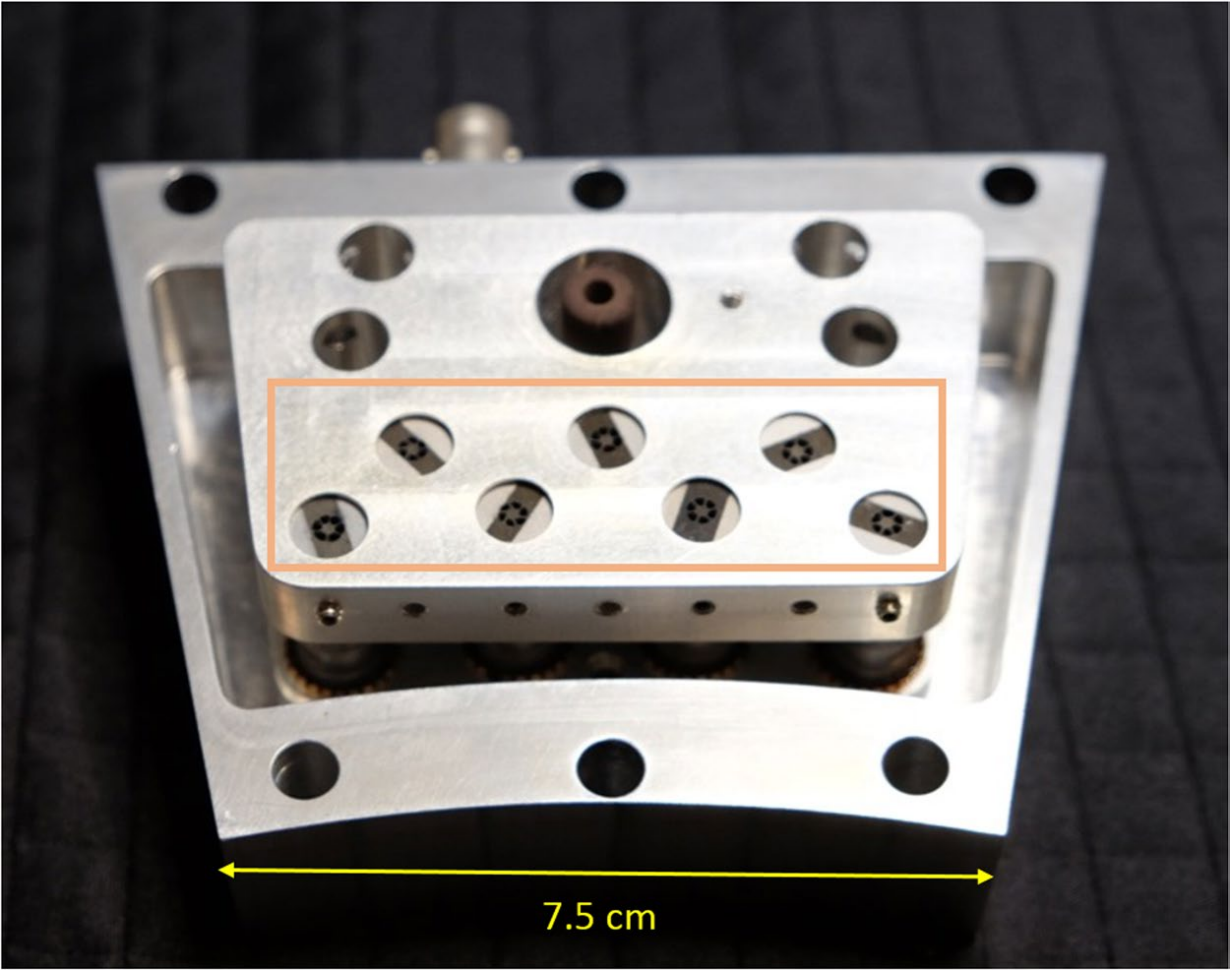
Internal view of the module, with the exit ports of the Channeltrons highlighted.

We constructed a module consisting of an arc of seven of these photocathode-based x-ray elements inside of a vacuum manifold (Figs 2 and 3). The module spans 24 degrees of an arc, with the inner circle measuring 227.5 mm in radius. The seven x-ray elements span the middle 17.5 degrees of this arc. A full ring of these modules would have 15 modules, an outer diameter of 57.0 cm and inner diameter of 38.8 cm. Such a source ring may be coupled with an adjacent concentric ring of detector arcs or flat-panel detectors. The x-ray sources are digitally controlled by the UV LED illumination using a microcontroller mounted on top of the x-ray source module.

The generated x-rays exit the vacuum housing though a beryllium window, directly adjacent to the anode. Beryllium was chosen for its low atomic number (z = 4), and relative stability in atmosphere.

A vacuum flange on the rear of the module allows for the housing to be connected to a turbopump, which maintains a vacuum of 10^−7^ Torr. A valve allows the vacuum to be maintained even when separated from the turbopump. The vacuum manifold also has a getter for maintaining vacuum allowing the option for it to be sealed off permanently once the desired vacuum is reached.

### X-ray Source Characteristics

The projected focal spot size measured on the x-ray detector for our source was approximately 1 mm × 4.5 mm. As mentioned above, each source is capable of approximately 1 mA of tube current. Source stability, another important requirement for tomographic imaging, was also measured and can be described in terms of: (1) stability of the photon flux in the X-ray beam, and (2) stability of the beam spectrum.

Our experiments reveal that the X-ray flux, which is proportional to the tube current, is stable over the duty cycle for which the sources are intended to be used. In our experience, the beam current can be assumed to be the same from pulse to pulse when the x-ray tube is used for up to several minutes. Over longer periods of operation, with our source, as with most X-ray sources, the X-ray flux does change, primarily because of tube heating. To account for this effect, all CT systems (including ours) have an “air calibration” step where the un-attenuated X-ray beam is first measured and then used for normalizing each projection.

A second aspect of x-ray flux stability involves source to source variation in our multi-source setup. While there is a variability of up to a few hundred microamps in the beam current produced by each of the 7 sources for a given bias voltage and input illumination, this variation can be compensated for by adjusting the illumination and/or duty cycle of each source. Our custom-built controller board allows us to control the frequency, duty cycle, and luminance of the pulsed UV LED illumination of each x-ray element in order to achieve a uniform x-ray output from each source.

In order to assess the spectral stability, we measured the spectrum using an Amptek Cd-Te X-ray spectrometer (X123, Amptek Inc.). We found the beam spectrum, which is reflective of the tube voltage and atomic composition of the anode, to be stable throughout imaging. Since the atomic composition of the anode does not change, the beam spectrum is primarily a function of the stability of the power supply used for controlling the anode voltage. Extremely low ripple power supplies are available, and this parameter for our source is no different from any other X-ray source.

### Reliability

The proposed distributed source is highly reliable because of the life-time of the metallic photocathodes, the reliability of the electron amplifiers, the large thermal mass of the anode and the fact that it is stationary. The magnesium photocathode will continue to work as long as it is not exposed to air for long durations. The Channeltrons used in our design are rated to work for 10,000 hours in spectroscopy applications. Since we operate at high vacuum and the only input are electrons (rather than molecular ions in spectroscopy), we expect an even longer lifetime. In our experience, in over a year of testing and production, the only components to have broken are ceramic screws in the housing. Finally, if the source was sealed, as opposed to being actively pumped, one may have to re-establish the vacuum if it were to decline over time.

### Tomographic Imaging

**Figure 4:**
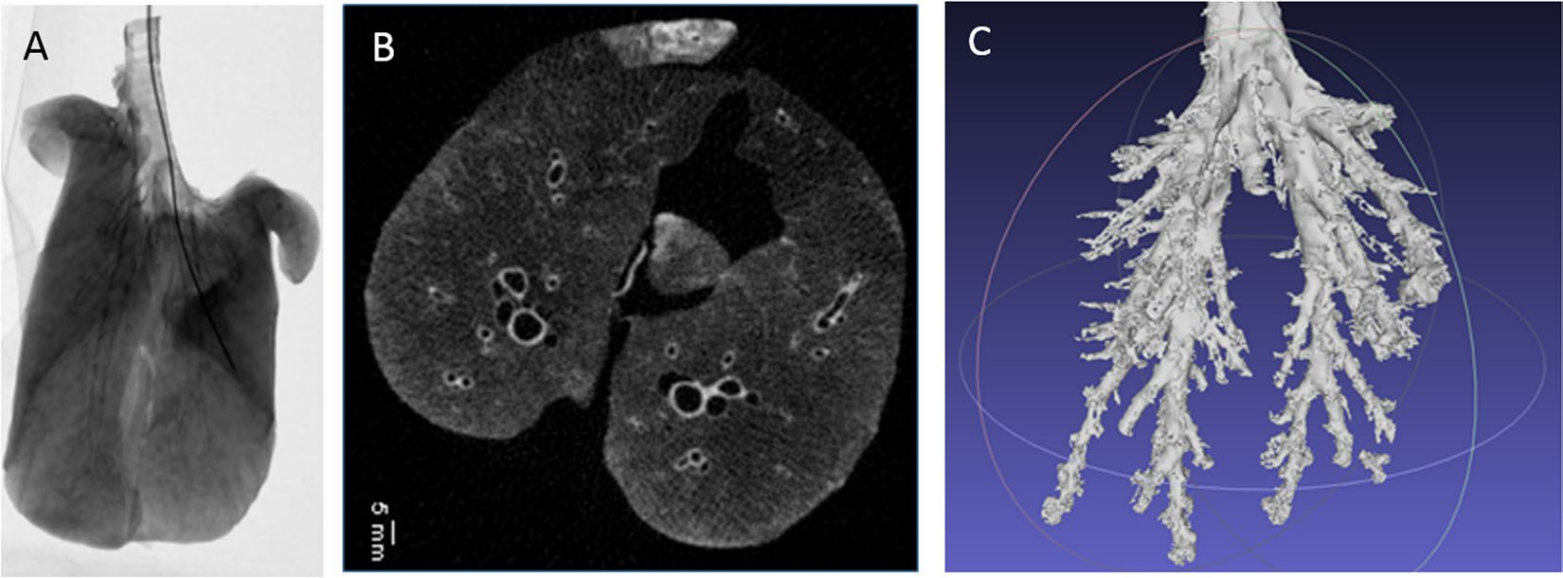
(**A**) X-ray projection image of pig lung with an inserted catheter. (**B**) Axial slice from pig lung reconstruction. (**C**) Segmented bronchial tree from pig lung.

Using a clinical flat-panel detector, we acquired fluoroscopic projection images of a catheter being inserted into the bronchioles of a pig lung (Fig. 4A). In order to acquire tomographic images, we simulated a full 360-degree ring of x-ray sources using a rotation stage for the specimen. For each position of the rotation stage, seven images, one from each x-ray element, were acquired at a tube voltage of 35 kVp. We implemented a Filtered Back Projection (FBP) reconstruction algorithm to create 3D volumetric images (Fig. 4B,C) from a series of projection images.

The projection images were acquired using a Varian flat panel detector in 2 × 2 binning mode, for an effective pixel size of 278 microns. The voxel size of the reconstructed pig lung images was 0.45 mm, isotropic. The minimum resolvable feature size in the reported images was 0.6 millimetres. These parameters are quite competitive with state-of-art CT scanners currently in use. We have imaged cadaveric hand, wrist, knee, bovine lung specimens and qualitative comparison by a radiologist (R.G.) found the imaging to be of comparable image quality.

### Scaling-up for Full-body Tomography

The current prototype operates at 40 kVp or less. However, this is only a limitation of the current prototype and can be overcome by increasing the tube voltage to 120 kVp or higher, a number that is more typical of human scanning. Such a scale-up will increase the size of our source. However, as detailed below, a 3-fold increase in tube voltage does not severely impact the size, weight and portability of our design.

**Figure 5:**
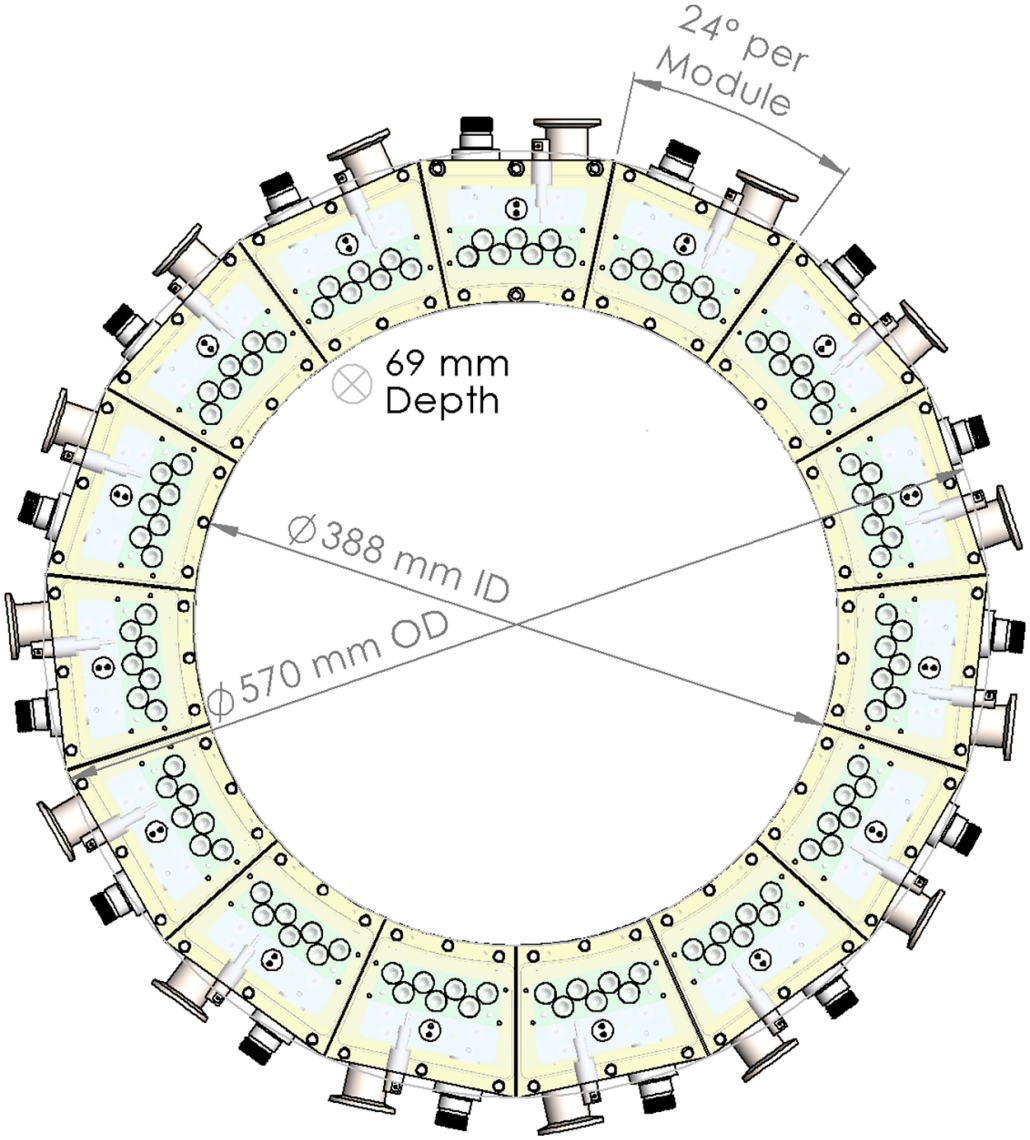
CAD diagram of full ring of modules, with dimensions.

Figure 5 shows a schematic diagram of the system gantry with the key distances marked. Increasing the tube voltage to 120 kVp will necessitate proportionate increase in the distance of the anode (the high voltage component) from the cathode and the housing, specifically the X-ray window. We are currently replacing the HV connection with a candlestick type connector and re-designing the vacuum manifold such that the critical distance between the key components is suitable for high voltage operation. The separation between the anode and the x-ray window and between the anode and rear wall (the critical dimensions) are each about 15% of the total front-to-back length of the module. To support 120 kV operation, these distances would have to be tripled, increasing the total length by a factor of 1.3. As calculated in our CAD designs, this would increase the weight by approximately 40%, to 1.5 kg per module. Therefore, re-designing our distributed source for voltages traditionally used for medical imaging does not detract from its scalability, portability, and the other advantages described above.

For comparison, a conventional CT system can weigh over 2000 kilograms, without the patient bed, and occupy a volume of 228 (h) × 188 (w) × 156 (d) cm to provide a bore size of 70 to 80 cm. Even specially designed “portable” neuroimaging CT scanners are nearly 500 kilograms and 153 (h) × 134 (w) × 72 (d) cm for a bore size of 32 cm^33,34^.

For any CT scanner designed for human imaging, the size of the bore is fixed by the anatomy (i.e., head-only, full-body, or extremity-only scanner) and cannot be decreased. Also, the requirements on the patient table are unchanged by the scanner design, as are the requirements on reconstruction computer and power supply. The static CT presented here, however, does decrease the overall weight and size of the scanner because of the following factors: (1) it simplifies the system gantry; (2) it removes the motor and the gear system for rotating thousands of kilograms of mass; (3) it eliminates the high-bandwidth slip ring required for transferring the projection data from the rotating components to the static computer; (4) being motion-free, it simplifies the high-voltage distribution on the gantry; and finally, (5) it eliminates the rotating anode and the elaborate cooling system that is mandated by the high wattage - up to 100 kW or more for a general-purpose, high-end scanner - being dumped at a single millimetre sized focal spot on the anode.

The size and weight implications of these changes are significant. As shown in Fig. 5, the dimension of the overall gantry with the current 7-element modules - a ring that is suitable for a head-only CT scanner - can be thought of as a donut with a 38.8 cm bore, 57.0 cm outer diameter, 6.9 cm depth, and an estimated weight of 22.5 kg. These specifications are considerably more attractive than the current state-of-art 3rd generation scanners.

While difficult to estimate and compare the cost implications of the above-mentioned simplifications, we expect the static CT system to be cheaper because it does not need a slip ring, a rotating gantry, and a tube cooling system.

The overall shielding requirements for the proposed system are no different from those of a conventional CT system. However, given the static nature of our design, the points in space where the radiation is being generated, and the volume that will be irradiated, are fixed in space and time. As a result, one can design a tailored shielding scheme that is built into the distributed ring of sources. Such internal shielding, which can be quite close to the points of x-ray generation, can be designed to minimize size and weight, a task that we have currently not undertaken.

In regard to scalability and portability, it should be mentioned that scaled-down versions of the traditional rotating CT scanners are available for portable deployment^33^. These scanners typically reduce the bore size of the gantry to a head-only scanner. By virtue of the inverse-square law, the x-ray source power increases as the square of the bore size. Therefore, reducing the bore-size by approximately a factor of 3 results in an order of magnitude decrease in the x-ray source power required for imaging, with concomitant reduction in weight and cooling requirements. The rotational mechanism, along with its power train and slip ring for data transfer, however, persists in these designs. As portability is accomplished by trading off bore size, these designs are not scalable: any increase in bore size, for example to accommodate the whole body, will bring us back to the original heavy, non-portable system. This is well illustrated by the portable full-body CT scanners currently available (e.g., the BodyTom^TM ^^34^). These so-called portable scanners, which weigh as much as any other conventional scanner, should be regarded as a traditional CT scanner with a motorized base for in-hospital transportation rather than as a portable scanner for field deployment.

## Discussion

Our prototype demonstrates several specific advantages that could enable CT imaging in austere environments. Timing of the UV LEDs, rather than current modulation, controls the x-ray flux. The system has no moving parts and each module weighs approximately 1 kilogram. Individual CT modules could be stored and transported in a compact storage box and then assembled in space or in the field for operation. When not in use, the CT system can be disassembled and stored away. Modules can be brought up to atmospheric pressure for repairs and replacement of individual source elements.

In current CT scanners, it is customary to modulate the tube current in response to the changing attenuation as the scanner rotates around a patient. With individually addressable sources that can be pulsed at different duty cycles, it is possible to further reduce radiation dose by selective illumination.

The compact, modular construction of our system would make it easier to combine it with positron emission tomography (PET) for simultaneous PET/CT imaging. Since our system dispenses with the slip-ring and all other moving parts in a CT gantry, tighter coupling between the CT and the PET stages of a hybrid PET/CT scanner would be feasible. Since a rotating gantry cannot be easily combined with the high magnetic field of a Magnetic Resonance scanner, our static CT system opens the door for a portable simultaneous MR/CT system which will have clear applications in trauma, stroke, and cancer imaging.

There are several opportunities for improvement in future generations of our current prototype, which currently has several limitations that include: the lack of electron beam focusing lens, the low beam current, and a voltage ceiling of 40 kVp. Of these limitations, only the beam current is inherent to the use of a photocathode for electron generation. We are working to redesign the Channeltrons so that they have a thinner profile and incorporate an Einzel lensing system consisting of a series of electrostatic lenses at the output of the Channeltron.

In conventional CT, the image quality is dependent on the integrated photon flux over one rotation. As the rotation time (seconds or s) decreases, the tube current (mA) must proportionally increase in order to maintain the product mA × s, which provides a measure of the delivered dose. The fastest rotation one can achieve in conventional CT (and accordingly, the minimum scan time) is governed by mechanical considerations with the current state-of-the-art limited to about 5 rotations per second. Beyond this rotation speed, a gantry weighing thousands of kilograms generates insurmountable centripetal force on the x-ray tube, electronics, and other rotating components.

In switching from a mechanical rotation of a 3rd generation CT scanner to the digital control of the proposed stationary CT, we eliminate a fundamental barrier to faster CT scans. However, even though the mechanical rotation is no longer involved, one still needs to accumulate the requisite photon flux to achieve a given image quality. Higher photon flux for each element in our distributed X-ray source can be achieved by (1) increasing the active surface area of the electron multipliers, (2) optimizing the photocathode deposition process, (3) increasing the multiplier bias voltage, and (4) replacing the 255 nm UV LEDs with a switched 180 nm UV lamp. In addition, even though each source in static CT is weak, there are many of them and they can be arbitrarily and simultaneously turned on or off to provide any coded pattern. The patterns that can be employed for scanning, and the algorithms that can be used to reconstruct tomographic images from these coded patterns, are a topic of ongoing research.

If sufficient flux is achieved, increasing the pulse rate could further reduce the scan times. Currently, the slowest component limiting the pulse rate of the x-ray sources is the set of optoisolators separating the 5 V logic and 20 V power lines on the LED control circuit (see Methods). These have a rise time of 6 microseconds. A faster pulse rate would allow for faster scan times in tomographic imaging. If the x-ray pulse rate were able to be reduced to the order of nanoseconds (a capability well within the reach of a metallic photocathode, which has a sub-picosecond response time) time-domain x-ray imaging - with the potential for a dramatic reduction in delivered dose - is feasible^35^. Such time-domain imaging would also allow many recent advances from the LIDAR community to be applied to x-ray imaging.

Reducing the cost of the bill of materials would be an important step in bringing our prototype to the clinic, and would help ensure that it is available to the billions of patients around the world in middle- and low- income communities. One possibility in particular that holds promise is using cheaper LEDs with a broader spectrum and poorer heat management. A broad spectrum is of no particular concern to us, but heat sinking the LEDs and isolating them thermally from the electron amplifiers (which have a highly heat-dependent behavior) would become an important task.

In acquiring tomographic images with a series of miniaturized, photocathode-based sources, we have demonstrated a novel method of volumetric x-ray imaging, and a fundamental re-imagining of x-ray tomography. We have also demonstrated the utility of a metallic photocathode as the electron generation method for x-ray imaging.

## Methods

### Magnesium Deposition

The magnesium deposition was carried out using a thermal evaporator (Denton Vacuum systems, 505-A). Fifty mg pellets of magnesium were placed in a tungsten wire basket. The magnesium was vaporized by running 25 amps of current through the tungsten wire for 2 minutes, under vacuum (<10^−5^ Torr). Deposition occurred inside of a Pyrex bell jar pumped down to vacuum using a liquid nitrogen cooled diffusion pump. Foreline pressure on the diffusion pump was maintained with a mechanical roughing pump. Individual Channeltrons were wrapped in aluminum foil and held to a rotating stage underneath the tungsten basket with carbon tape. Alongside the Channeltron, a glass microscope slide was placed on the rotation stage to confirm successful deposition.

The chamber (commonly used for gold and carbon coating) was first extensively cleaned to remove the possibility of any contamination with other materials. No other metals (besides magnesium) were used in the chamber after the cleaning process. Cleaning was accomplished in a 3 step process- the chamber was disassembled, and each component scoured with acetone and 800 grit sandpaper. The process was repeated a second time with isopropyl alcohol (IPA) instead of acetone. Finally, each component in the chamber was scrubbed using IPA and Kimwipes.

A bias voltage of between 3000 and 4000 volts is applied across the Channeltron. An adjustable high voltage between 10 and 40 kV is applied from the exit of the Channeltron to the tungsten anode target. The weak electron beam produced by illuminating the magnesium-coated input of the Channeltron with UV light is multiplied up to 10^8^ times by the Channeltron. The 10–40 kV potential difference between the Channelton output window and the anode accelerates the electrons from the Channeltron and imparts upon them the energy which is partially converted into x-ray photons at the anode by the Bremsstrahlung process.

### UV LED control

We designed a printed circuit board (PCB) to control the 7 UV LEDs. The PCB has two separate circuits: a 20 V power line and a 5 V logic line. The circuit uses a board-mounted Arduino Micro to control the pulses of the UV LEDs, through a bi-polar junction transistor (BJT). The 5 V logic lines are kept on a protected ground and separated from the 20 V power lines by optoisolators. A constant current is provided to the LEDs by two constant current diodes (CCRs) in parallel. These units limit the current to a maximum of 100 mA to protect the expensive LEDs, which are limited to 100 mA. However, the current provided by the CCRs can be adjusted by a potentiometer in series with one of the CCRs. A visible indicator LED is also in parallel with each UV LED. A high-side current sense chip (LT1787) provides feedback from each LED circuit to the Arduino, and allows us to monitor in real time the response times and current draw of the UV LED. 4-pin phoenix connectors on the top of the board enable transmission and reception of timing signals from the flat-panel detector and a Thorlabs PRM1Z8 rotation stage. The PCB layout was designed using DipTrace, an E-CAD software. The PCB itself was manufactured by Imagineering, Inc.

### High Voltage Controls

The x-ray source is powered and monitored by an integrated, custom built high voltage system, and controlled by a LabView script. In addition to setting and regulating the high-voltage power flowing to the targets and the multipliers, the system has built-in HV fault handling and data logging capabilities. The high-voltage hardware in the unit is controlled via a LabJack UE9 digital/analog IO device. Separate high-voltage power supplies (HVPS) are used to maintain the 10–40 kV anode voltage (Matashusada XRT-505) and the 3 kV bias on the electron amplifiers (EMCO F30). In addition to the HV power supplies, the high-voltage system contains three relay boards and three low-voltage power supplies (LVPS). One of the LVPS provides 24 V to the anode HVPS, and another provides 12 V to the amplifier HVPS. The final LVPS provides power for the relays. The relays are controlled through the output ports of the Labjack, a programmable 0–5 V output. Two relays control the high-voltage outputs, and a third controls the interlock of the anode 25–50 kV HVPS. This HVPS also requires a set-point input, which is provided by amplifying the 0–5 V Labjack output to 0–10 V. Current and voltage measurements are recorded using the analog input channels on the Labjack.

The LabView VI controlling the high voltage system is designed to be easy to use and to fail safe in the event of arcing. The user interface allows voltage control as well as individual control over the relays in the system. Further, it shows the power supply parameters (actual voltage and current) every 100 ms and logs them. It includes features such as overvoltage shutdown, automatic safe-mode in case of interrupted communications, a 10s-watchdog timer and automated data logging. A 50 KΩ ballast resistor, immersed in mineral oil, was added to the output of the power supply to reduce a significant electrical arcing problem. With the ballast resistor, we are able to achieve continuous operation at up to 40 kV. Higher voltages will require a significant redesign of the housing module to increase the critical dimensions. The aluminum housing has an SHV connector for the multiplier bias, and a silicone connector/cable (MPF, Inc.) for the 10–40 kV line.

### Anode targets

The tungsten anode rods are held in place by set screws. For ease of machining, we used tungsten carbide rods that were slightly alloyed with cobalt and iron.The tungsten rods are set at a 10 degree angle to the Channeltrons, and thus the electron beam. This reduces the projected spot size in one dimension, creating an ellipse.

### Geometric Calibration

We are able to obtain the geometric information necessary for image reconstruction in a single acquisition using a custom designed cylindrical 3D-printed phantom. The phantom contains 14 ball bearings embedded in a thin cylinder of ABS plastic. To perform the calibration, images are acquired on the flat-panel detector as the phantom is rotated through different angles for each x-ray source. By tracking the position of the ball bearings as the phantom is rotated, the distances from each source to the detector plane and rotational axis can be determined.

### Image Acquisition

Imaging acquisition was done using a Varex Imaging 2530DX flat panel detector, which has a 278 um^2^ pixel size in 2 × 2 binning mode. The panel was synchronized with the control board. After the completion and installation of 7 miniature x-ray sources, we were able to acquire volumetric images of several biological samples. Processing of the volumetric images was performed using 3D Slicer.

### Safety and Handling

Vaporized magnesium is a respiratory irritant, and powdered magnesium can oxidize rapidly in water. Diffusion pump oil (used in the thermal evaporation system) is also a respiratory irritant, and the diffusion pump must be cryogen-cooled to prevent back-streaming of the pump oil.
